# The evolutionarily conserved genes: *Tex37*, *Ccdc73, Prss55* and *Nxt2* are dispensable for fertility in mice

**DOI:** 10.1038/s41598-018-23176-x

**Published:** 2018-03-21

**Authors:** Manan Khan, Nazish Jabeen, Teka Khan, Hafiz Muhammad Jafar Hussain, Asim Ali, Ranjha Khan, Long Jiang, Tao Li, Qizhao Tao, Xingxia Zhang, Hao Yin, Changping Yu, Xiaohua Jiang, Qinghua Shi

**Affiliations:** USTC-SDJH Joint Center for Human Reproduction and Genetics, The CAS Key Laboratory of Innate Immunity and Chronic Diseases, Hefei National Laboratory for Physical Sciences at Microscale, School of Life Sciences, CAS Center for Excellence in Molecular Cell Science, University of Science and Technology of China (USTC), Collaborative Innovation Center of Genetics and Development, Hefei, 230027 Anhui China

## Abstract

There are more than 2300 genes that are predominantly expressed in mouse testes. The role of hundreds of these genes has been studied in mouse spermatogenesis but still there are many genes whose function is unknown. Gene knockout (KO) strategy in mice is widely used for *in vivo* study of gene function. The present study was designed to explore the function of the four genes: *Tex37, Ccdc73, Prss55 and Nxt2*, which were evolutionarily conserved in eutherians. We found that these genes had a testis-enriched expression pattern in mice except *Nxt2*. We knocked out these genes by CRISPR/Cas9 individually and found that all the KO mice had normal fertility with no detectable difference in testis/body weight ratios, epididymal sperm counts, as well as testicular and epididymal histology from wild type mice. Although these genes are evolutionarily conserved in eutherians including human and mouse, they are not individually essential for spermatogenesis, testis development and male fertility in mice in laboratory conditions. Our report of these fertile KO data could avoid the repetition and duplication of efforts which will help in prioritizing efforts to focus on genes that are indispensable for male reproduction.

## Introduction

The transmission of male heritance occurs through a highly-sophisticated process called spermatogenesis. During mammalian spermatogenesis, spermatogonia undergo mitosis to maintain their population and produce primary spermatocytes (2n). It is followed by the meiotic phase in which the haploid spermatids (n) are produced by two consecutive divisions. The spermatids undergo the process of spermiogenesis forming mature spermatozoa^1^. All these stages of spermatogenesis are spatio-temporally well-regulated and thousands of genes are involved in its successful completion.

It has been indicated that over 2300 genes are predominantly expressed in testes^2^. Most of these genes are believed to be essential for spermatogenesis or sperm function but the roles of many of these are still unknown. To study the specific function of such testis-enriched mammalian genes, gene knockout (KO) strategies are the efficient tools which have been used extensively^3–5^. The functional roles of more than 400 genes have been elaborated, with most of them being indispensable for male fertility^6–13^. However, a small number of genes have been studied to be dispensable for spermatogenesis and male fertility even though they have predominant testicular expression^14,15^. Indeed, the infertile KO mice are always reported but fertile KO mice are only published when the gene is well known.

Recently, Miyata *et al*. used various genome editing techniques to knockout 54 evolutionarily conserved and testis-enriched mouse genes. They did not find any essential roles of these genes in male reproduction^15^. They elaborated the importance of disseminating the fertile KO mice data to scientific community and academia, which can save valuable resources by preventing the same KO recapitulation^15^. Hence, it is vital to publicize such important data which would help researchers in prioritizing energies to focus on genes essential for male reproduction.

Here we selected five human genes (*TEX37, CCDC73, PRSS55*, *LYZL11* and *NXT2)* which have predominant testicular expression and are evolutionarily conserved in eutherians. We knocked out these genes using CRISPR/Cas9 system in mice. However, during the course of our work, *Lyzl1* KO mice were reported by Miyata *et al*.^15^. As the deletion region of our *Lyzl1*^−/−^ mice (c.[188_206del; 210_242del]/c.[188_206del; 210_242del]) was different from those reported by Miyata *et al*. (c.21_34del/c.21_34del)^15^, therefore, we still included the generation and analysis of *Lyzl1*^−/−^ mice as independent confirmation of the Miyata *et al*. data in our study. We did not observe any obvious defects in spermatogenesis and fertility in all these KO mice. Thus, our study revealed that, these selected genes are not individually vital for mouse spermatogenesis and fertility.

## Results

### Identification and phylogeny of the testis-enriched genes

**Figure 1:**
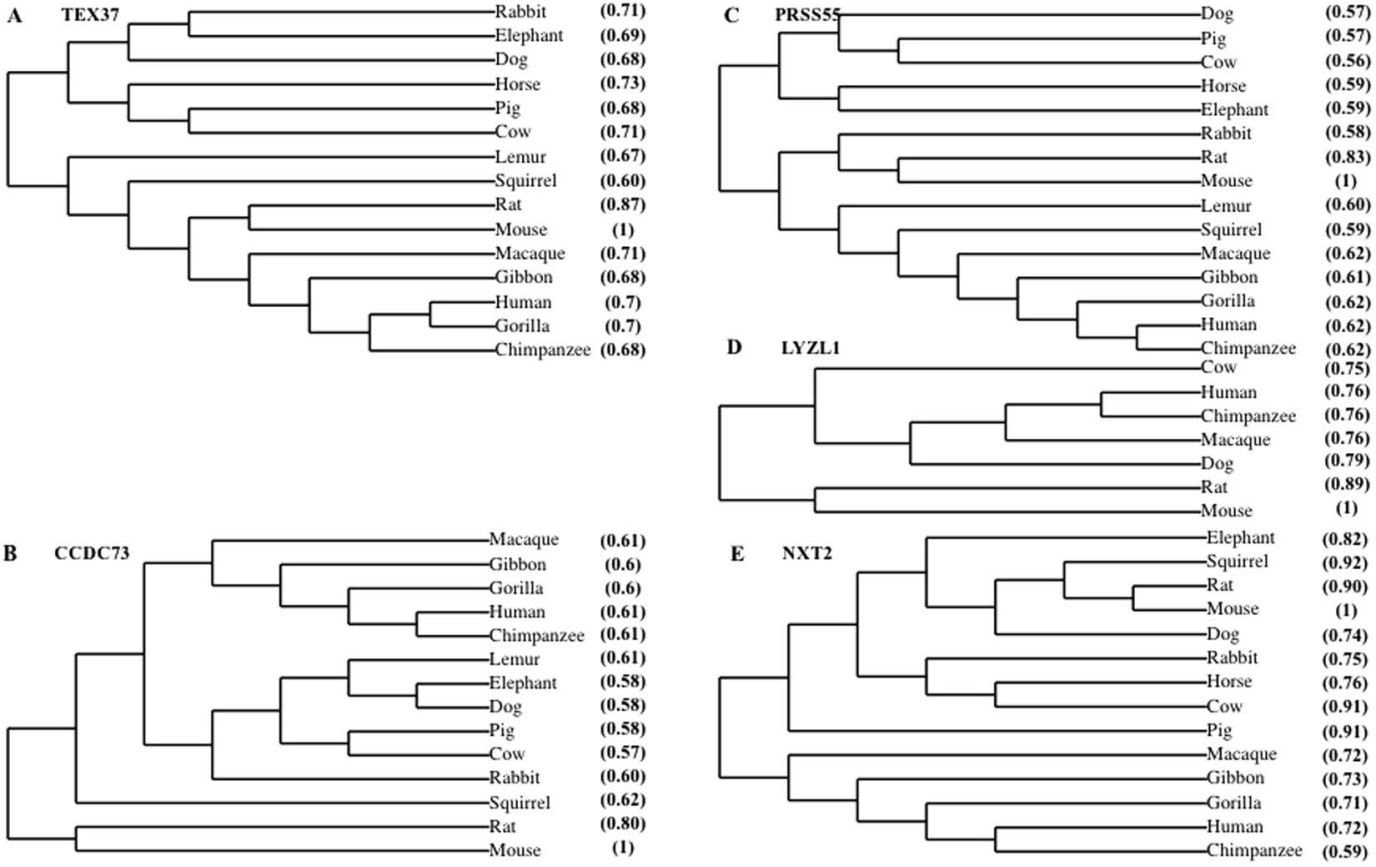
Conservation of selected genes in eutherians. Multiple proteins sequence alignments were performed by T-coffee. Phylogenetic trees were constructed using online database TreeDyn from multiple protein sequence alignment^36–40^. Phylogeny of (**A**) Tex37, (**B**) Ccdc73, (**C**) Prss55, (**D**) Lyzl1, and (**E**) Nxt2. Parentheses show percent identity to reference sequence (mouse:1).

**Figure 2:**
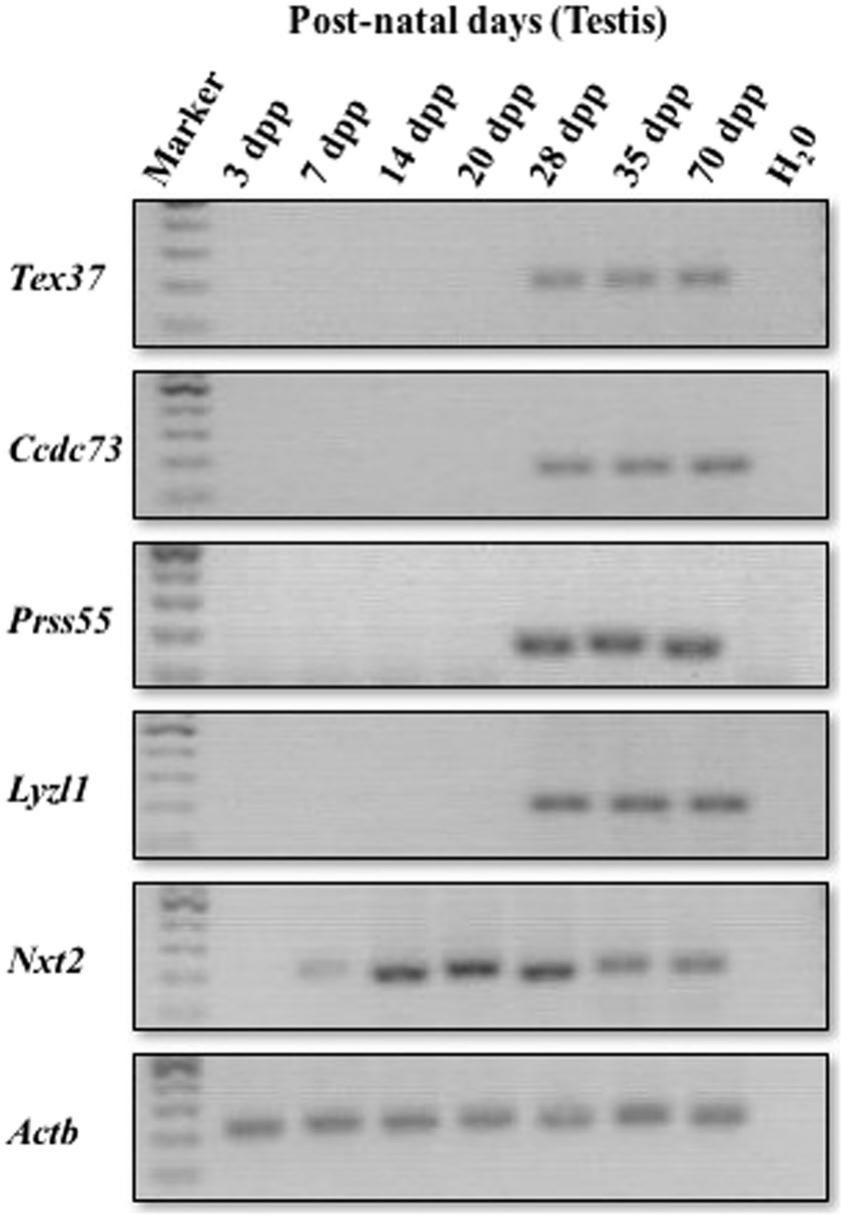
mRNA expression of the selected genes. Postnatal temporal expression of genes in testes of 3, 7, 14, 20, 28, 35 and 70-dpp-old mice was analyzed by RT-PCR. *Actb* was used as positive control. H_2_0 was used as negative control. Cropped gels are shown here. Full-length gels are provided for review in the supplementary Figure S2a.

Human genes with predominant testicular expression and conserved open reading frames (ORFs) in mouse were identified by NCBI searches (Table S1). We selected 5 human genes: *TEX37, CCDC73, PRSS55*, *LYZL11* and *NXT2* which were shown to be highly expressed in testes (Table S2). Phylogenetic analysis of their orthologs showed a higher level of sequence resemblance implying that the proteins are conserved in many of the eutherians (Fig. 1). In mouse, these selected genes also had testis-enriched expression except *Nxt2* which showed expression in other tissues based on NCBI searches (Table 1). We further carried out RT-PCR to check the temporal expression of these selected genes during postnatal testis development using testicular cDNAs from mice of different ages (Fig. 2). The findings revealed that *Nxt2* expression began in 1-week-old testes, which suggested its possible role in early spermatogenesis. Whereas *Tex37*, *Ccdc73*, *Prss55* and *Lyzl1* were initially expressed in 4-week-old testes, implying their possible role in late spermatogenesis (spermiogenesis) or fertilization.

**Table 1:** Expression of the selected genes in mouse tissues.

Tissue	*Tex37* (RPKM)	*Ccdc73* (RPKM)	*Prss55* (RPKM)	*Lyzl1* (RPKM)	*Nxt2* (RPKM)
Brain	0.00	1.87	0.00	0.00	1.98
Heart	0.00	0.22	0.00	0.00	1.08
Intestine large	0.00	0.05	0.00	0.00	1.69
Intestine small	0.00	0.00	0.00	0.00	0.13
Kidney	0.00	0.12	0.00	0.00	4.49
Liver	0.00	0.05	0.00	0.00	1.65
Lung	0.00	0.09	0.00	0.00	1.25
Mammary gland	0.00	0.07	0.00	0.00	0.89
Ovary	0.00	0.06	0.00	0.00	0.20
Placenta	0.00	0.64	0.00	0.00	1.07
Spleen	0.00	0.06	0.00	0.00	0.27
Stomach	0.00	0.00	0.00	0.00	0.09
Testis	105.01	80.39	127.78	49.15	0.56

RPKM: Reads per kilo base per million base mapped reads.

https://www.ncbi.nlm.nih.gov.

### Generation of KO mice

**Figure 3:**
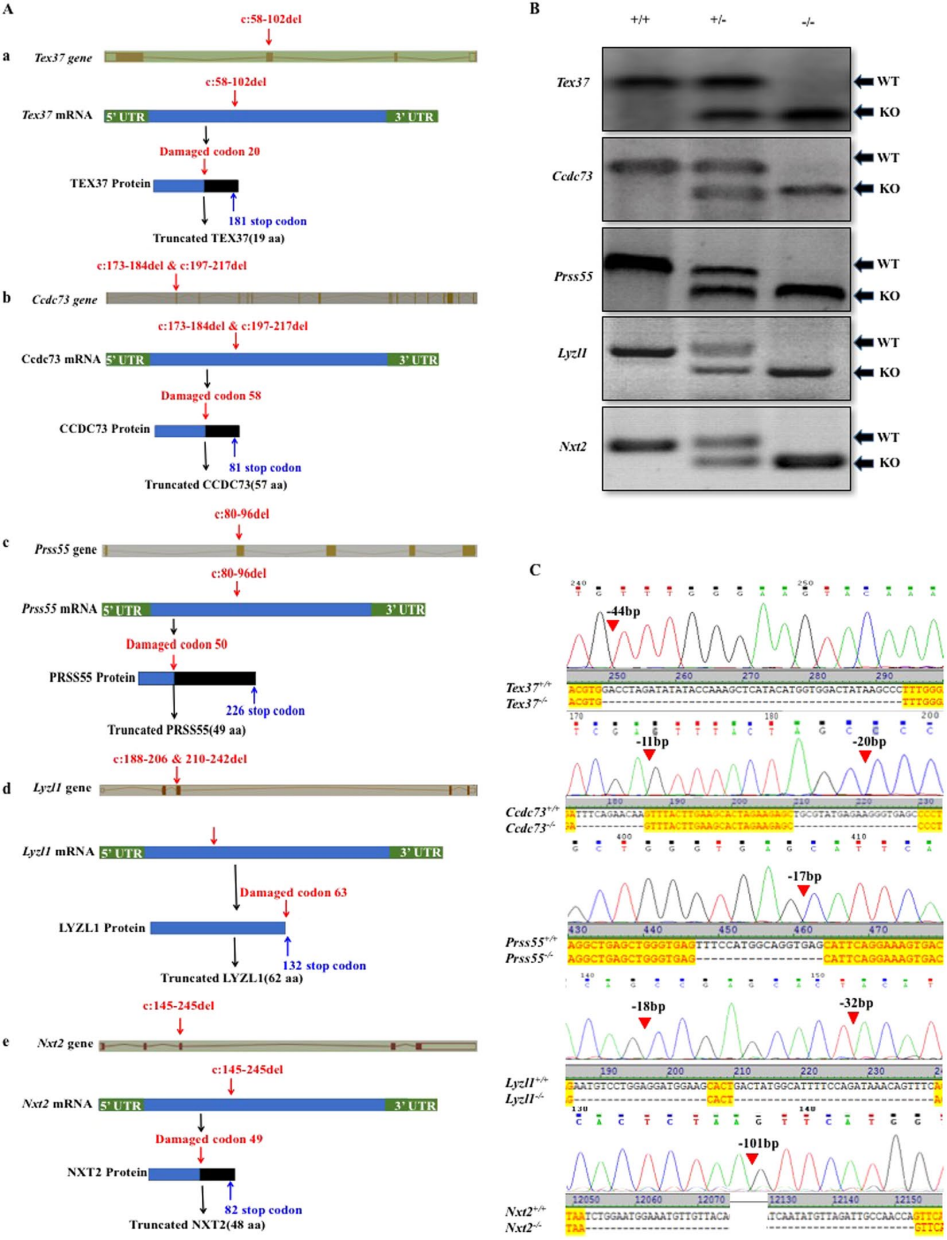
Knockout strategy and genotyping of mutant mice. (**A**) Schematic strategies for the generation of KO mice using CRISPR/Cas9. 44 bp of *Tex37* (a), 11 bp and 20 bp of *Ccdc73* (b) and 17 bp of *Prss55* (**c**) were deleted from Exon 2. While, 18 bp and 32 bp of *Lyzl1* (d) and 101 bp of *Nxt2* (e) were deleted from exon 3. (**B**) Genotype of each KO mouse was confirmed with PCR. (**C**) Representative Sanger sequence image for the verification of each KO mouse. Red arrow heads above the chromatograms and dashes in aligned cds sequences show the deletions.

To investigate the function of these 5 genes in a short span of time, KO mice were produced by CRISPR/Cas9 technique. To be noted, *Lyzl1* KO was previously reported by Miyata *et al*.^15^. Homozygous mice having large deletions forming frameshift mutations, were obtained by selective breeding (Fig. 3A). PCR and Sanger sequencing results confirmed the deletions of targeted regions (Fig. 3B,C). All the KO mouse lines exhibited normal development.

### Normal fertility and sperm count of the KO mice

**Figure 4:**
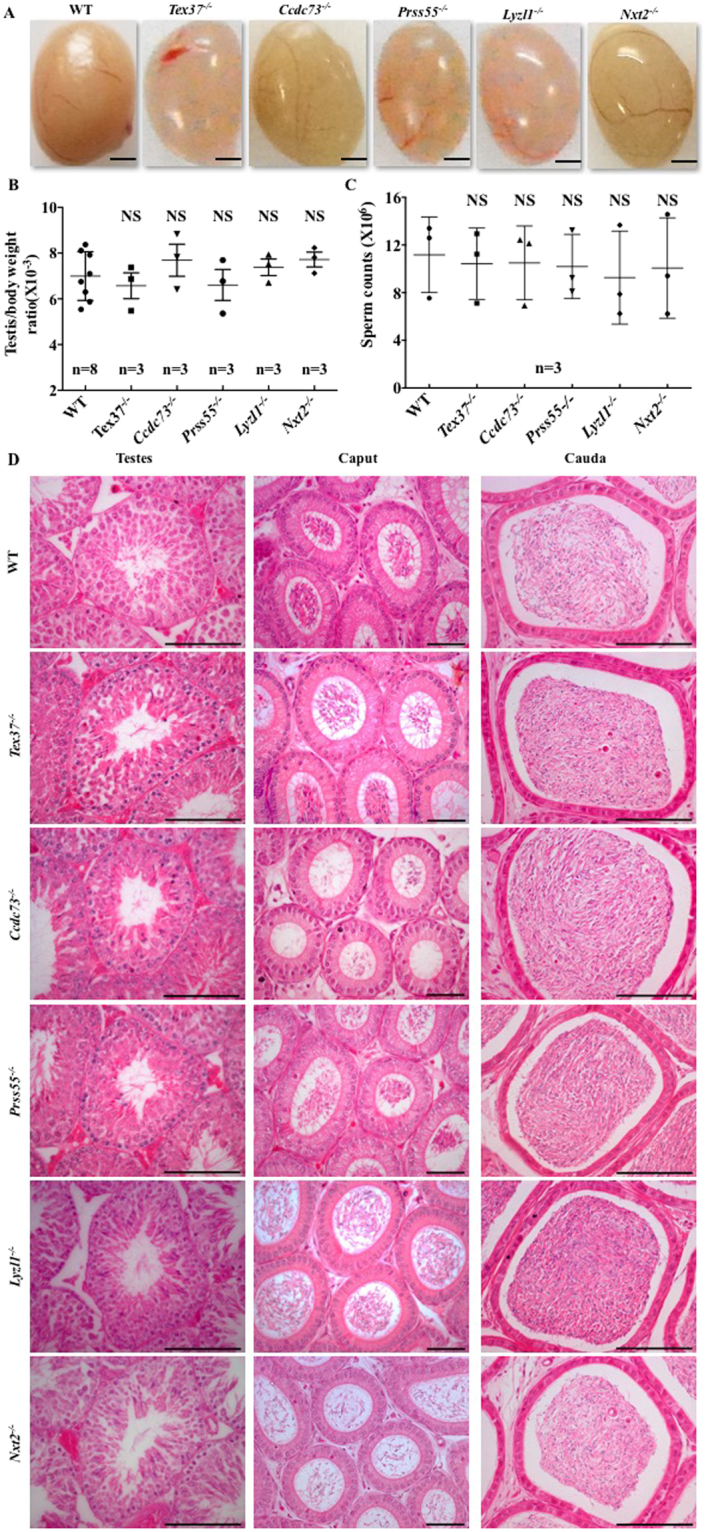
Fertility and spermatogenesis of the KO mice. (**A**) Representative images of testes from 70-dpp-old WT and KO mice. Scale bars, 2 mm. (**B**) Testis/body weight ratio of 70-dpp-old WT and KO mice. *Error bars* represent SD. n, the number of animals. NS, no significant difference, Student’s *t*-test was performed between WT and each KO mouse group for testis/body weight ratio. (**C**) Sperm count of 70-dpp-old WT and KO mice. *Error bars* represent SD. n, the number of animals. NS, no significant difference, Student’s *t*-test was performed between WT and each KO mouse group for sperm count. (**D**) H&E staining of testes and epididymides caput and cauda from 70-dpp-old WT and KO mice. Scale bars, 100μm. The data shown is representative images from at least three mice.

After the confirmation of successful gene deletions, we analyzed the fertility of each KO male by breeding it with wild type (WT) females. The mating behavior of the KO mice was similar to that of WT with 100% fertility. The average number of litters per male/month and the average number of pups per litter produced by WT and each KO male were comparable (Table 2). Next, the testes from adult WT and each KO mouse were inspected for morphology and testis/body weight ratio. The examination revealed no significant difference between the WT and each KO group (Fig. 4A,B). Similarly, no significant difference was observed for sperm counts between WT and each KO group (Fig. 4C). Finally, histological sections from WT and each KO testis and epididymides were assessed. The examination illustrated intact seminiferous tubules with normal spermatogenesis having spermatogenic cells from spermatogonia to spermatozoa, and abundant spermatozoa in epididymides of WT and KO mice (Fig. 4D). Altogether these results reflected that complete knockout of *Tex37, Ccdc73, Prss55*, *Lyzl1* and *Nxt2*, which are testis-expressed genes, have no detectable effects on spermatogenesis and fertility of mice under normal laboratory conditions.

**Table 2:** Fertility assay.

Genotype	Mating period (months)	No. of fertile males (%)	Average litters/male/month	Average pups/litter
WT	4	5 (100)	1.94 ± 0.24	7.52 ± 1.03
*Tex37* ^−/−^	2	2 (100)	1.75 ± 0.36^NS^	7.71 ± 2.36^NS^
*Ccdc73* ^−/−^	2	3 (100)	1.83 ± 0.29^NS^	6.82 ± 1.54^NS^
*Prss55* ^−/−^	4	4 (100)	1.94 ± 0.13^NS^	7.68 ± 1.01^NS^
*Lyzl1* ^−/−^	3	3 (100)	1.56 ± 0.19^NS^	6.86 ± 2.32^NS^
*Nxt2* ^−/−^	2	3 (100)	1.83 ± 0.29^NS^	7.45 ± 1.29^NS^

Each male was bred with two females. WT, wild type. Student’s *t* test was performed for average litters/male/month and average pups per litter between WT and each knockout mouse group. NS, no significant difference. Data are presented as mean ± SD.

## Discussion

According to the report of Schultz *et al*., 2300 mouse genes have predominant testicular expression^2^. Extensive work has been done on genomic and transcriptomic levels to determine the function of these genes. Still, the role of many of these genes needs to be elaborated, we thus selected the four mouse genes expressed in testis (*Tex37, Ccdc73, Prss55* and *Nxt2)* for the study of their functional roles in mouse spermatogenesis and fertility.

The five genes have conserved ORFs both in human and mouse (along with some other eutherians) with at least 75% identity in coding nucleotide and 60% identity in amino acid sequences (Table S1). These similarities suggested critical roles played by these genes during spermatogenesis and male fertility. From NCBI, we checked the expression of these genes in various mouse tissues which showed that all the genes had testis-enriched expression except *Nxt2* which have expression in other mouse tissues (Table 1). It has been reported that deficiency of the genes with similar expression profile, such as *Zmym3, Ku70*, *Fam46d*, *Pdha2*, *Tex101* and *Spata19*, etc. always resulted in spermatogenic problems leading to male infertility, thus validating the essential roles of these genes^16–18^.

Therefore, to figure out the functional roles of the selected genes, we generated homozygous knockout mice by CRISPR/Cas9. Using similar procedures by Miyata *et al*.^15^, we confirmed the disruption of targeted genes by PCR genotyping and Sanger sequencing (Fig. 3). The antibody was available only for TEX37, hence, we could only check TEX37 at protein level in the WT and *Tex37* KO mice. The examination further verified the successful deletion of targeted gene (Supplementary figure S1). Indeed, the absence of any alternate translation initiation site in the ORFs of disrupted genes excluded the possibility of any functional proteins produced from alternate translation initiation sites^19^. Moreover, our deletions were large enough that truncated protein, if formed, would be non-functional (Fig. 3C).

To determine the roles of each gene in male fertility, we compared the number (average litters/male/month) and size (average pups per litter) of litters produced by each KO group with those in WT males and found no significant difference. The *Lyzl1* KO mice have also been reported by Miyata *et al*. with their number and size of litters being comparable with the controls^15^, which is consistent with our observations. Although some KO mice with normal fertility but decreased testicular size and sperm count have been reported^20–22^, our KO mice were not only fertile but also exhibited normal testicular size and sperm count. The possible reasons for the lack of obvious phenotypes in these KO mice might be functional redundancy. In current scenario, deficiency of these five targeted genes would be compensated by other factors^23^. For example, there are several members in *Ccdc* family, thus the insufficiency of *Ccdc73* could be counteracted by other members, such as *Ccdc42*, which is necessary for proper sperm development^24^. Likewise, we determined that *Tex37* KO males are fertile, possibly its paralogs, *Tex11* and *Tex14*, may compensate its deficiency^25,26^. Similarly, *Nxt1* has testis enriched expression, presenting 75% of its amino acid sequence identity with NXT2, which implies that *Nxt1* might compensate for *Nxt2* deficiency. Thus, future studies on double/triple knockout animals for these genes with their paralogs may further enhance the understanding of their roles in testes and male fertility.

To be noted, we only analyzed the phenotype in normal laboratory conditions, we cannot exclude the possibility that these genes may be functional in some stimulated condition. Additionally, though all the KO mouse lines apparently exhibited normal development, some of these genes have expression at basal/low level in tissue(s) other than testis, therefore, the possible existence of unnoticed phenotype(s) cannot be excluded. Thus, we could only conclude that *Tex37*, *Ccdc73*, *Prss55*, *Lyzl1* and *Nxt2* are dispensable for fertility of male mice in the same genetic backgrounds under normal laboratory conditions.

In summary, we generated KO mice of the genes *Tex37, Ccdc73, Prss55*, *Lyzl1* and *Nxt2* which have testis-enriched expression in human and are conserved across most of the eutherian species. These KO mice exhibited normal fertility, and testicular development and function. As the fertile *Lyzl1* KO mice have been reported previously by Miyata *et al*.^15^, our results further identified four new genes role in spermatogenesis. These results not only indicate that these genes do not play a prominent role in mouse fertility, but also help scientific community, reproductive biologists and academia to focus on genes indispensable for testis development and male fertility.

## Materials and Methods

### RNA extraction and RT-PCR

We performed RNA extraction and RT-PCR as explained previously^27,28^. TRIzol reagents (Takara, 9109) were used for the extraction of total RNAs. cDNAs were synthesized by using the PrimeScript RT kit (TaKaRa, RR047A) from total RNAs as described in manufacturer’s procedure. EasyTaq DNA Polymerase (TransGen Biotech, AP111) was used to perform RT-PCR with the following cycle conditions: 94 °C for 5 minutes, following 35 cycles; each at 98 °C for 15 seconds, 53 °C for 30 seconds, 72 °C for 20 seconds and finally, 72 °C for 2 minutes. RT-PCR was performed with total volume of 30 μl PCR mix by adding 15 μl of 10xEasy Taq DNA Polymerase buffer (Trans, AP111), 1.8 μl of 2.5 mM dNTPs (Trans, AD101), 0.8 μl of reverse and forward PCR primers (10 μM), 0.3 μl of Easy Taq DNA Polymerase (Trans, AP111) and 2 μl of template cDNA. RT-PCR for the selected genes and *Actb* (used as positive control) was performed simultaneously and gel electrophoresis was run in parallel.

### Generation of KO mice

KO mice were produced by one-cell embryo injection using the CRISPR/Cas9 genome editing strategy as reported^29^. The second or third exon of genes were targeted by two guide RNAs (Table S3). We designed single guide RNAs (sgRNAs) targeting a specific exon as described previously and selected these sgRNAs on the basis of rare off-target hits using computational predictions by CRISPRdirect (https://crispr.dbcls.jp) or the Bowtie software^30–32^. After *in vitro* transcription of sgRNAs, they were co-injected with Cas9 mRNA into zygotes of B6D2F1 (C57BL/6 × DBA/2 J) mice and then embryos were transferred to pseudo pregnant ICR females^30^. After Sanger sequencing verification, the male and female founders carrying a heterozygous (^+/−^) deletion mutation were inbred to produce homozygous (^−/−^) mice. These mice were fed with food and ddH_2_O *ad libitum* and held in specified photoperiod (lights on 08:00–20:00) in the laboratory animal center of University of Science and Technology of China (USTC). All the procedures and analyses on laboratory animals were conducted according to the institutional guidelines which were approved by Institutional Animal Care Committee of USTC.

### Fertility test

For fertility testing, each 10 to 12-week-old WT or KO male was separately housed with two sexually mature WT C57BL/6 females for indicated mating periods (Table 2). The number of litters and pups from each pregnant female was recorded at birth.

### Hematoxylin and eosin (H&E) staining

The 70-day-old WT and KO mice were euthanized by cervical dislocation. After the removal, testes and epididymides were instantly fixed overnight in Bouin’s solution. The tissues embedding were carried out in paraffin for block formation. Tissue sections were prepared by microtome and subsequently H&E staining was performed as described previously^33^. The experiments were repeated thrice by using samples from different sets of mouse testes. To keep the inter-experimental deviations minimum, all the procedures on WT and knockout testes were carried out concurrently. Digital Nikon DS-Ri1 camera installed on a Nikon Eclipse 80i microscope was used to capture images.

### Sperm counting

For sperm counting, 70-day-old mice were euthanized, their epididymides were isolated and followed by multiple incisions. Sperm were released in 1 ml buffer after incubation of these epididymides for 30 minutes at 37 °C in a CO_2_ (5%) incubator. This buffer comprised of NaCl (75 mM), EDTA (24 mM), and 0.4% bovine serum albumin (Sigma, A2058). Sperm were collected from this buffer solution by nylon-mesh filtration. Subsequently hemocytometer was used for sperm counting as reported previously^33^.

### Western blot

Testicular lysates were prepared in lysis buffer containing NaCl (300 mM), EDTA (5 mM), Tris/HCl (50 mM) with pH of 7.4, Triton X-100 (1%) and protease inhibitors (Roche, 04693116001, Basel, Switzerland). Western blot was then performed followed by band quantification as outlined^34,35^. Primary antibodies for TEX37 (1:500; Protein tech, 25464-1-AP, USA) and Glyceraldehyde-3-phosphate dehydrogenase (GAPDH; 1:1000; Millipore, MAB374, MA, USA) were used.

## Electronic supplementary material

Supplementary File
