# Supplementary material for: The evolutionarily conserved genes: Tex37, Ccdc73, Prss55 and Nxt2 are dispensable for fertility in mice
Source: Sci Rep. 2018 Mar 21;8:4975. doi: 10.1038/s41598-018-23176-x (PMC5862965; doi:10.1038/s41598-018-23176-x)
Supplement: Supplementary file 1 — Supplementary File [file 41598_2018_23176_MOESM1_ESM.docx]

**The evolutionarily conserved genes: *Tex37*, *Ccdc73*, *Prss55* and *Nxt2* are dispensable for fertility in mice**

Manan Khan^1, #^, Nazish Jabeen^1, #^, Teka Khan^1^, Hafiz Muhammad Jafar Hussain^1^, Asim Ali^1^, Ranjha Khan^1^, Long Jiang^1^, Tao Li^1^, Qizhao Tao^1^, Xingxia Zhang^1^, Hao Yin^1^, Changping Yu^1^, Xiaohua Jiang^1*^, Qinghua Shi^1*^

**Figure S1**. **Confirmation of Tex37 deletion in KO mice.**


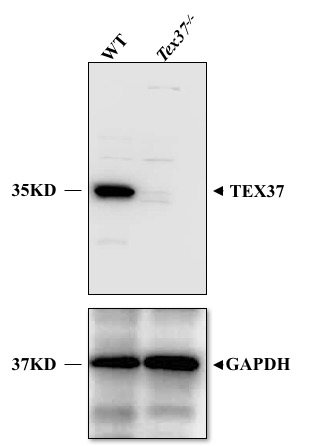


**Figure S1**. **Confirmation of Tex37 deletion in KO mice.** Testes from 70-dpp control and *Tex37* KO mice were collected for Western blot analysis of Tex37. Anti-Tex37 polyclonal antibody recognized the band near 35kDa in the WT but not in KO mice, indicating that Tex37 is indeed deleted. GAPDH was served as protein loading control. The image shown here is representative of three independent experiments.

**Figure S2a. Supplementary full length cDNA gel.**

**
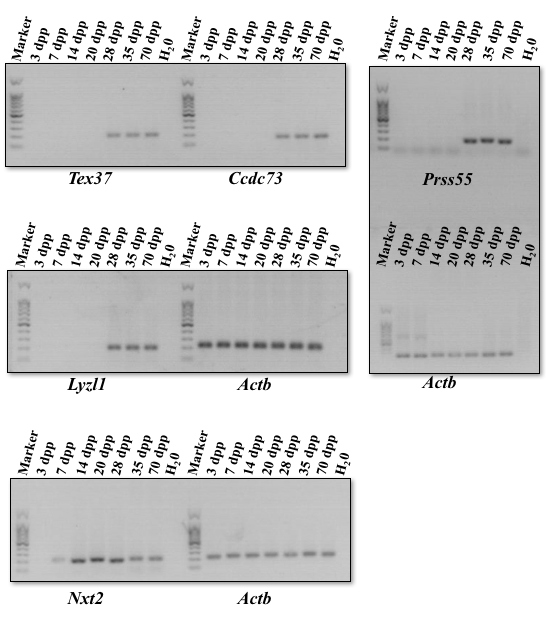
**

**Figure S2a. Supplementary full length cDNA gel.** Postnatal temporal expression of genes in testes of 3, 7, 14, 20, 28, 35 and 70-dpp-old mice was analyzed by RT-PCR. *Actb* was used as positive control. H_2_O was used as negative control. RT-PCR for the selected genes and *Actb* (used as positive control) was performed simultaneously and gel electrophoresis was run in parallel.

**Table S1: Genes with conserved ORFs and protein sequences in human and mouse**

| **Gene** | **Chromosomal location** | **cDNA size** | **Number of exons** | **Protein size** | **MGI** | **Identical human-mouse CDS** | **Identical human-mouse protein** |
| --- | --- | --- | --- | --- | --- | --- | --- |
| ***Tex37*** | **Chr6:70913087-70918927 bp, - strand** | **814 bp** | **Exons:4, Coding exons:3** | **180 residues** | [**MGI:1921471**](http://www.informatics.jax.org/marker/MGI:1921471) | **79%** | **70%** |
| ***Ccdc73*** | **Chr2:104867805-105017904 bp, + strand** | **3,944 bp** | **Exons:18, Coding exons:17** | **1,066 residues** | [**MGI:3606488**](http://www.informatics.jax.org/marker/MGI:3606488) | **76%** | **61%** |
| ***Prss55*** | **Chr14:64075438-64090162 bp, - strand** | **1,032 bp** | **Exons:5, Coding exons:5** | **321 residues** | [**MGI:1918287**](http://www.informatics.jax.org/marker/MGI:1918287) | **75%** | **62%** |
| ***Lyzl1*** | **Chr18:4165832-4182232 bp, + strand** | **846 bp** | **Exons:5, Coding exons:4** | **148 residues** | [**MGI:1914578**](http://www.informatics.jax.org/marker/MGI:1914578) | **84%** | **76%** |
| ***Nxt2*** | **ChrX:142226770-142239692 bp, + strand** | **2,532 bp** | **Exons:5, Coding exons:5** | **198 residues** | [**MGI:2147914**](http://www.informatics.jax.org/marker/MGI:2147914) | **88%** | **72%** |

**Table S2. Expression of the selected genes in human tissues**

| **Tissue** | ***TEX37***  **(RPKM)** | ***CCDC73***  **(RPKM)** | ***PRSS55***  **(RPKM)** | ***LYZL1***  **(RPKM)** | ***NXT2***  **(RPKM)** |
| --- | --- | --- | --- | --- | --- |
| **Brain** | **0.00** | **2.28 ± 0.30** | **0.00** | **0.00** | **3.78 ± 2.48** |
| **Heart** | **0.00** | **1.24 ± 0.14** | **0.00** | **0.00** | **0.77 ± 0.18** |
| **Intestine small** | **0.00** | **1.16 ± 0.10** | **0.00** | **0.00** | **2.92 ± 0.26** |
| **Kidney** | **0.00** | **1.99 ± 0.18** | **0.00** | **0.00** | **2.71 ± 0.29** |
| **Liver** | **0.00** | **1.09 ± 0.15** | **0.00** | **0.00** | **1.97 ± 0.35** |
| **Lung** | **0.00** | **1.49 ± 0.16** | **0.00** | **0.00** | **3.54 ± 0.4** |
| **Ovary** | **0.00** | **3.78 ± 0.44** | **0.00** | **0.00** | **2.34 ± 0.07** |
| **Placenta** | **0.00** | **1.50 ± 0.05** | **0.00** | **0.00** | **3.74 ± 0.60** |
| **Spleen** | **0.00** | **1.99 ± 0.25** | **0.00** | **0.00** | **2.76 ± 0.28** |
| **Stomach** | **0.00** | **1.17 ± 0.11** | **0.00** | **0.00** | **2.87 ± 0.60** |
| **Testis** | **20.42 ± 12.67** | **4.23 ± 0.63** | **4.40 ± 1.74** | **12.40 ± 4.20** | **16.76 ± 4.67** |

**RPKM: Reads per kilo base per million base mapped reads**

[**https://www.ncbi.nlm.nih.gov**](https://www.ncbi.nlm.nih.gov)

**Table S3. sgRNAs used for CRISPR/Cas9 system**

| **Gene** | **sg1-Top** | **sg1-Bottom** | **sg2-Top** | **sg2-Bottom** |
| --- | --- | --- | --- | --- |
| ***Tex37*** | **TAGGTATATATCTAGGTCCACGTGG** | **AAACCCACGTGGACCTAGATATATA** | **TAGGTGGACTATAAGCCCTTTGGG** | **AAACCCCAAAGGGCTTATAGTCCA** |
| ***Ccdc73*** | **CGATTTCAGAACAAGTTTAC** | **GTAAACTTGTTCTGAAATCG** | **TAGAAGAGCTGCGTATGAGA** | **TCTCATACGCAGCTCTTCTA** |
| ***Prss55*** | **GCTGGGTGAGTTTCCATGGCAGG** | **CCTGCCATGGAAACTCACCCAGC** | **CCATTTCTGCGGCGGCTCCATTC** | **GAATGGAGCCGCCGCAGAAATGG** |
| ***Lyzl1*** | **TAGGCACTACAGCCGAGAATGTCC** | **AAACGGACATTCTCGGCTGTAGTG** | **TAGGATCAGAAACAGTACCTGAGC** | **AAACGCTCAGGTACTGTTTCTGAT** |
| ***Nxt2*** | **CAAAGCCACTCTAATCTGG** | **CCAGATTAGAGTGGCTTTG** | **ACCAGTTCATGGTGAGTATC** | **GATACTCACCATGAACTGGT** |

**Table S4. Primers used for RT-PCR**

| **Gene** | **Primer forward** | **Primer reverse** | **Anneal temperature (°C)** | **Band size (bp)** |
| --- | --- | --- | --- | --- |
| ***Tex37*** | **TCAGCTCCAGAGCAAAGAGTTTT** | **AAGCATAATGGCAGGTGGTG** | **53** | **191** |
| ***Ccdc73*** | **GAAGTAAAAGACAAGCTGTGC** | **GGATGCTTTGTTACTTCGAG** | **53** | **189** |
| ***Prss55*** | **GCAGGTGAGCATTCAGGAAA** | **GCCGTTTAAAGCCTTTGTGC** | **53** | **206** |
| ***Lyzl1*** | **GAAGTCTGTTGGTGTCTTCGC** | **GACATTCTCGGCTGTAGTGTT** | **53** | **190** |
| ***Nxt2*** | **GTAGAGCTGCCGAGGAATTTG** | **CACTGGTCACAACGAGCACT** | **55** | **249** |
| ***Actb*** | **AGGCTGTGCTGTCCCTGTAT** | **CTCTCAGCTGTGGTGGTGAA** | **53** | **208** |

**Table S5. Primers used for genotyping**

| **Gene** | **Primer forward** | **Primer reverse** | **Anneal temperature (°C)** | **Band size (bp)** |
| --- | --- | --- | --- | --- |
| ***Tex37*** | **GAAAGTGACATTCGACAGGG** | **TCTTAGGGACACAGCAACTG** | **55** | **324** |
| ***Ccdc73*** | **TGAACTTGGAGAGTTATGGG** | **AGCCATGCATACATGATGAG** | **57** | **447** |
| ***Prss55*** | **TTAGGATGACAAGCCCATGC** | **TCTTCCTCCCACTGTTTGTC** | **55** | **812** |
| ***Lyzl1*** | **ACCCTGATCCTGTACACATC** | **CCTGCTGAAAACTGGAATGC** | **57** | **563** |
| ***Nxt2*** | **ATGCAGCATGGAGAGTCCTC** | **GAAGACCCAAAGCCTGACTG** | **55** | **432** |
